# Impaired Leptin Signalling in Obesity: Is Leptin a New Thermolipokine?

**DOI:** 10.3390/ijms22126445

**Published:** 2021-06-16

**Authors:** Valentina Annamaria Genchi, Rossella D’Oria, Giuseppe Palma, Cristina Caccioppoli, Angelo Cignarelli, Annalisa Natalicchio, Luigi Laviola, Francesco Giorgino, Sebastio Perrini

**Affiliations:** Section of Internal Medicine, Endocrinology, Andrology and Metabolic Diseases, Department of Emergency and Organ Transplantation, University of Bari Aldo Moro, 70124 Bari, Italy; valentina.genchi@uniba.it (V.A.G.); rossella.doria@uniba.it (R.D.); g.palma7@studenti.uniba.it (G.P.); cristina.caccioppoli@uniba.it (C.C.); angelo.cignarelli@uniba.it (A.C.); annalisa.natalicchio@uniba.it (A.N.); luigi.laviola@uniba.it (L.L.); francesco.giorgino@uniba.it (F.G.)

**Keywords:** leptin, thermogenesis, obesity, leptin-resistance, adiposity, hyperleptinaemia, brown adipose tissue

## Abstract

Leptin is a principal adipose-derived hormone mostly implicated in the regulation of energy balance through the activation of anorexigenic neuronal pathways. Comprehensive studies have established that the maintenance of certain concentrations of circulating leptin is essential to avoid an imbalance in nutrient intake. Indeed, genetic modifications of the leptin/leptin receptor axis and the obesogenic environment may induce changes in leptin levels or action in a manner that accelerates metabolic dysfunctions, resulting in a hyperphagic status and adipose tissue expansion. As a result, a vicious cycle begins wherein hyperleptinaemia and leptin resistance occur, in turn leading to increased food intake and fat enlargement, which is followed by leptin overproduction. In addition, in the context of obesity, a defective thermoregulatory response is associated with impaired leptin signalling overall within the ventromedial nucleus of the hypothalamus. These recent findings highlight the role of leptin in the regulation of adaptive thermogenesis, thus suggesting leptin to be potentially considered as a new thermolipokine. This review provides new insight into the link between obesity, hyperleptinaemia, leptin resistance and leptin deficiency, focusing on the ability to restore leptin sensitiveness by way of enhanced thermogenic responses and highlighting novel anti-obesity therapeutic strategies.

## 1. Introduction

Obesity is considered a chronic medical condition whose pathogenesis has a multifactorial origin. Environment factors, feeding habits, nutrient quality, psychosocial variables and genetic background altogether are known to cooperate in the onset of adiposity and related metabolic disturbances [1,2]. When an imbalance between energy consumption and disposal occurs, adipose tissues lose their ability to reservoir fatty acids, thus promoting lipid spillover into ectopic organs. To avoid these unfavourable metabolic consequences, a plethora of endocrine hormones are normally released to preserve energy homeostasis. Among these factors, leptin represents the main adipocytokine able to generate healthy metabolic processes principally via neuronal circuits. Leptin acts as an energy-level signaller whose secretion is reduced in a fasting state and increased after nutrient intake. In particular, leptin stimulates several hypothalamic nuclei, thus promoting satiety and body weight reduction [3]. Therefore, a lack of leptin release or impaired leptin signalling leads to overnutrition, energy expenditure (EE) reduction and the development of an obesogenic phenotype and other chronic diseases [4]. Similarly, under energy overload, the organism accumulates lipid excess in the adipose tissue, resulting in fat enlargement and hyperleptinaemia. Indeed, circulating levels of leptin are proportional to the adipose tissue mass [5] and, thus, the enhanced secretion of leptin might be a consequence of obesity. Long-term exposure to leptin overload, however, can also result in a still ill-defined state of ‘leptin resistance’ wherein abnormal leptin receptor (LEPR) activity is observed. In this scenario, leptin-dependent anorexic effects are lacking in the presence of persistent leptin stimulation due to the existence of a potent feedback mechanism that relies on the induced suppression of cytokine signalling 3 (SOCS3) and protein tyrosine phosphatase (PTP1B) expression, resulting in a blocked leptin signalling cascade [6].

Accordingly, a vicious cycle begins, since the failure of leptin signalling induced by hyperleptinaemia reduces the satiety control, thus favouring overfeeding, which, in turn, increases leptin secretion. Nevertheless, not all obesity conditions are the result of LEPR dysfunctions. Current evidence has revealed several genetic determinants, such as single-nucleotide polymorphisms (SNPs), which affect leptin gene expression and whose incidence increases the risk of developing overweight and obese conditions.

Beyond regulating feeding behaviours and catabolic responses through its neuroendocrine activities, leptin has been recently considered a ‘thermolipokine’ since it appears to support both thermogenic and browning responses. In particular, leptin may promote brown adipose tissue (BAT) activity, sustaining a heat balance via central mechanisms [7,8,9,10]. Although some in vivo and in vitro studies have established the autocrine effects of leptin on adipose tissue biology (i.e., adipogenesis, lipogenesis) [11,12,13], few to date have verified the direct action of this hormone on BAT functions.

In accordance with the pleiotropic actions of leptin, emerging studies have evaluated the efficacy of leptin-based and LEPR antagonist therapy as anti-obesity approaches in the setting of both leptin sensitivity and resistance states. Herein, we will discuss current knowledge regarding the emerging thermogenic role of leptin and related mechanisms in an obesogenic environment, as well as the effects of potentiating leptin signalling as an anti-adiposity strategy.

## 2. Genetics of Leptin

The human leptin gene (*LEP*) is localised on the 7alpha31.3 chromosome and is structured by three exons and two introns [14]. *LEP* encodes a peptide hormone consisting of 167 residues, which is primarily secreted by the adipose tissue into the bloodstream. Leptin reaches several brainstem areas, including the hypothalamus (i.e., arcuate nucleus), supporting glucose and energy balance control through the activation of LEPRs [15].

Nevertheless, the expression and secretion of leptin might be impaired under specific genetic backgrounds, thus promoting the onset of adiposity and related metabolic disturbances. Approximately 5% of cases of severe early-onset obesity are attributable to monogenic forms, which particularly affect both *LEP* and *LEPR* [16,17], whose diagnosis and identification are essential to improving care management. Currently, only eight different mutations in *LEP* that are thought to cause severe obesity have been reported [18].

**Figure 1:**
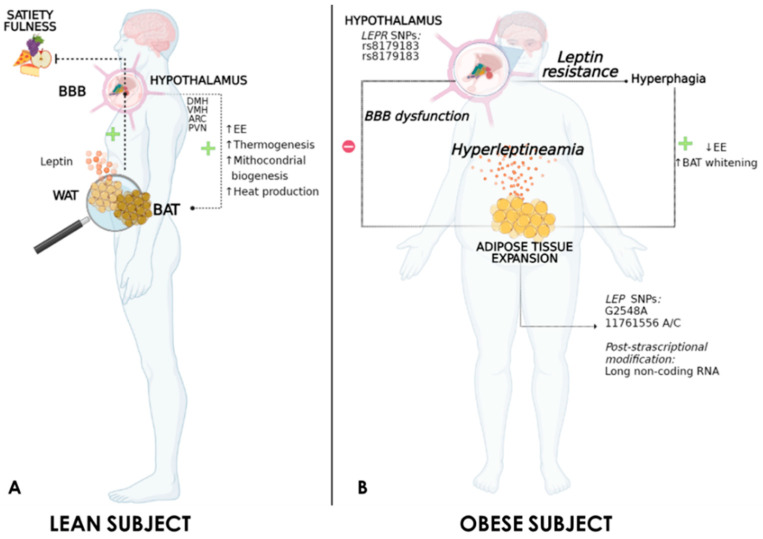
Possible relationship between circulating leptin levels and leptin sensitivity in lean (**A**) and obese (**B**) subjects. ↑ increase, ↓ decrease; + activation, − inhibition.

In the last few decades, an increasing body of evidence has documented higher prevalence rates of common obesity, which may be due to an interplay between environmental determinants (i.e., dietary habits, sedentary lifestyle, socioeconomic conditions) and individual genetic predisposition [19]. In particular, genome-wide association studies have investigated the influence of specific genetic loci in the pathophysiology of obesity, identifying different *LEP* and *LEPR* polymorphisms and measuring their impacts on adiposity development (Figure 1) [20]. For instance, findings concerning the *LEP* G2548A gene variant include several controversial results. *LEP* G2548A polymorphism is known to affect the expression of leptin, particularly at the transcriptional level, thus determining modifications of its adipose secretion [21]. The presence of allele G was found to be associated with body mass index (BMI) and serum leptin, regardless of ethnicity [22,23,24] or sex [25,26], even though recent advances reported that obesity status and female sex might exert modifying effects on polymorphism-related leptin concentrations [27]. In accordance, when the frequency of allele G of *LEP* was correlated with anthropometric and metabolic parameters (e.g., BMI, waist and hip circumference, fasting blood glucose, serum leptin), a greater risk for obesity in both female children and adolescents was noted [25], suggesting that this variant might influence one’s susceptibility to metabolic disturbances and obesity already early on in life. Conversely, 2548-AA or AG carriers have significantly higher circulating leptin levels as compared with 2548-GG carriers, as observed in both Turkish and French populations [28,29]. More recent results from a meta-analysis performed on 1372 obese individuals (BMI > 30 kg/m^2^) and 1616 controls, however, concluded that an association between obesity and *LEP* G2548A polymorphism did not exist [30].

*LEP* G2548A is not the only variant implicated in the development of obesity and related metabolic derangements. Indeed, a recent genotyping analysis discovered a novel SNP within 3’UTR of the *LEP* gene. Nesrine et al. observed that *LEP 11761556* AC polymorphism was linked to higher leptin levels and a greater risk of developing obesity as compared with the AA genotype in Tunisian volunteers [31]. Given these studies, there is a paucity of homogeneity among genotyping data suggesting that both ethnic differences and sample size may affect the correlation results between LEP variants and obesity-related parameters.

In this scenario, the aberrant expression of LEPR also has a crucial role in the onset of both rare and common forms of obesity. The human *LEPR* gene encodes a single membrane-spanning receptor of the class I cytokine receptor family that consists of two splice variants whose long isoform is known to regulate leptin signalling, facilitating energy and feeding control [32,33]. When the expression of LEPR was truncated, both humans and mice developed hyperphagia and an obese phenotype [34,35]. Similarly, the presence of certain SNPs within *LEPR*, especially in the gene region coding regulatory and receptor-activation domains (e.g., rs8179183 and rs8179183) (Figure 1) [36], has been associated with both overweight and severe obesity [20]. Moreover, emerging data from genome-wide association studies revealed that several genetic variants of *LEPR* were associated with obesity development. In particular, Foucan et al. explored the effects of the K109R, Q223R and K656N variants of *LEPR*, highlighting a strong association between obesity, metabolic syndrome and serum leptin concentrations in the Afro-Caribbean population of Guadeloupe Island [37]. Nevertheless, the phenotypic heterogeneity of obesity caused by dysregulated LEPR may underestimate the contribution of other variants not completely known whose allelic frequencies change according to ethnic group [33].

Despite that genetic investigations have reported the effects of gene allelic variants of *LEP* on leptin release, the mechanisms underlying the regulation of its expression are still poorly investigated. For example, recent in vivo results have demonstrated that defects in leptin production might also be the result of post-transcriptional alterations (Figure 1). Particularly, whole-genome sequencing performed in three types of adipocytes (e.g., subcutaneous, visceral, brown) from diet-induced obese (DIO) mice allowed researchers to identify 68 regulated long-noncoding RNAs (lncRNAs), including Lnc-Leptin, whose expression increases according to adipogenesis [38] and whose knockdown leads to a reduction in leptin expression concomitantly with impaired adipocyte commitment as observed both in vitro and in vivo [38]. Interestingly, this lncRNA did not participate in the regulation of the basal expression of leptin but instead served as a metabolic sensor to regulate the expression of LEP upon various energy statuses in adipocytes [38]. Although several genetic analyses continue to provide evidence regarding the polymorphism that compromises the LEP/LEPR axis in an obesity setting, there are no comprehensive studies exploring the effects of SNPs on EE and thermoregulation.

## 3. Leptin and the Regulation of Thermogenesis

The maintenance of whole-body energy disposal is a tightly regulated process that involves a plethora of endocrine and neuronal factors, among which leptin represents a key mediator of the adipose tissue—brain axis [3]. Although the adipose-related metabolic responses evoked by leptin have been discussed, the understanding of the role of this adipose-derived hormone in the regulation of the thermogenic activity of BAT to date is still inadequate. In this regard, few and conflicting results to date have been obtained through in vivo approaches.

Several studies have ascribed an indirect thermogenic activity of leptin through the activation of the hypothalamic nuclei, where LEPRs are mainly expressed. When leptin was administered in vivo into both the dorsomedial [39] and ventromedial hypothalamus [40], increases in the body temperature and BAT activity were observed, along with the stimulation of LEPR in the same brain areas [41]. These effects, however, appeared to occur largely together with the release of catecholamines since both the sympathetic denervation of BAT [40] and β_3_-receptor antagonists [41] inhibited the leptin-related thermogenic responses.

Leptin-sensing neurons involved in the regulation of energy dissipation were also identified within the hypothalamic arcuate nuclei (ARCs). Indeed, the ablation of leptin signalling in ARCs produced blunted thermogenic responses and BAT activation [42]. However, when LEPR was in vivo abrogated in proopiomelanocortin (POMC) neurons of ARCs, which are well-known to foster fullness and satiety responses, mice developed mild obesity, hyperleptinaemia and glucose intolerance without changes in food consumption or EE [43,44,45,46,47,48].

The regulation of heat production by leptin could also be mediated via paraventricular nuclei (PVN) [49]. Indeed, an in vivo study observed that mice with genetic abrogation of *LEPR* from PVN had decreased core body temperatures and levels of EE when housed at room temperature and lacked cold-induced adaptive thermogenesis in association with weakened expression and activity of uncoupling protein 1 (UCP-1) in BAT [10].

In this scenario, leptin may favour a heat balance by regulating the plasticity of the sympathetic architecture of the adipose tissue. Wang et al. recently reported that, following exposure to chronic leptin treatment, obese mice with leptin resistance showed a restored sympathetic innervation in both white adipose tissue (WAT) and BAT via brain-derived neurotrophic factor neurons of PVN, which are also known to regulate EE [49]. Indeed, leptin-induced activation of brain-derived neurotrophic factor networks in mice facilitated an increased density of postganglionic sympathetic neurons, innervating fat tissue and ultimately helping to restore thermoregulation and lipid turnover in terms of increased protein and mRNA levels of UCP-1 in BAT and lipolysis in WAT as compared with in-control littermates [49]. The results of these studies confirm the important role of central leptin signalling in the regulation of body temperature homeostasis.

Nevertheless, while several data obtained on thermogenesis induction in response to direct injections of leptin into the intracerebroventricular clearly indicate that leptin is able to induce EE and also sympathetic outflow to BAT [39,40,41,49], other studies seem to deny the effect of leptin in the regulation of body temperature. Fischer et al. suggest that this discrepancy could derive from misleading and erroneous normalisations of EE [50]. They claim that the potential thermoregulatory control ascribed to leptin by in vivo models (i.e., cold tolerance, BAT activity, neuronal pathways) could result from an inappropriate interpretation of findings. Specifically, these authors interpreted the results indicating hypometabolism in the leptin-deficient *ob/ob* mice were due to a misleading calculation artefact resulting from expression of EE per gram of body weight and not per intact organism [50,51]. In this setting, when leptin was administered, mice increased body temperature probably by a reduction of heat loss through tail vasoconstriction without showing a thermogenic response in BAT. The authors concluded that the increase of body temperature observed after prolonged leptin infusion is not a thermogenic response, but rather a pyrexic increase in body temperature rather without the recruitment of BAT [51].

The browning process of WAT is another way by which the body controls temperature excursions. This phenomenon consists of the progression of white adipocytes toward a brown phenotype whose extent is strongly regulated by different neuronal peptides (e.g., catecholamine, norepinephrine) [8]. Leptin participates in this scenario by stimulating sympathetic arborisation and tone [9]. Indeed, leptin stimulates WAT browning via the activation of PI3K signalling within POMC neurons of ARCs, in turn increasing EE and leanness [52,53]. In this setting, leptin does not appear to act alone but instead synergistically with insulin. Dodd et al. demonstrated that when leptin and insulin were in vivo co-infused, an optimal process of central-induced WAT browning was observed [52].

Nevertheless, a negative role of leptin in the regulation of white-to-brown transdifferentiation has recently emerged. This result might be secondary to hypoglycaemia and hypoinsulinaemia related to a leptin-induced fasting period. In particular, under negative energy balance, the sympathetic outflow close to adipose tissue changes enough to warrant energy conservation [54]. Hence, leptin might indirectly participate, determining the degree of inhibition of both thermogenic and browning processes. The gap in the data regarding the direct actions of leptin in BAT biology has been filled by recent experimental advances. Wang et al. observed that in obese mice, leptin promoted a reduction in adipose tissue weight and directly reduced the lipid droplet size of isolated white adipocytes through inhibition of the Hedgehog (Hh) pathway, whose activity is known to have anti-browning effects [55]. In particular, leptin upregulated the expression of browning genes (e.g., *PGC-1α*, *PDRM16*, *UCP-1*) and increased the mitochondrial DNA content in association with a reduction in glioma-related gene (Gli) expression, a key effector of the Hh pathway [55].

Taken together, these results provide evidence that the thermogenic capability of leptin remains elusive, since some studies show that this lipokine could induce the thermogenic and browning phenomena by rebuilding sympathetic architecture and tone close to the adipose tissue, while other observation suggest that the thermoregulation by leptin could be accounted for by indirect effects, such as a pyrexic response.

## 4. Obesity-Associated Hyperleptinaemia and Leptin Resistance

As described above, obesity is a chronic condition that results from an imbalance between energy intake and EE and leads to many disabilities and comorbidities, including hypertension, dyslipidaemia, insulin resistance and inflammation [56,57], which in turn constitute important risk factors for the development of type 2 diabetes, cardiovascular diseases and different types of cancer [56,58].

A growing body of evidence has demonstrated that increased adipose tissue mass contributes directly to an increase in circulating levels of leptin; thus, most common forms of obesity are characterised by hyperleptinaemia and by leptin resistance, since pharmacological doses of leptin are unable to suppress food intake and body weight [59]. However, some authors introduced the concept of ‘selective leptin resistance’ in 2002 to explain how leptin might increase the blood pressure in obese individuals [60], although the net effects of hyperleptinaemia on cardiovascular diseases are still not clearly understood.

Two potential overlapping mechanisms of ‘selective leptin resistance’ have been proposed as follows: (1) differential leptin molecular signalling pathways exist that mediate selective as opposed to universal leptin action and (2) brain-site-specific leptin action and resistance occur [61]. Further considerations should be given to the possibility that other physiologically and clinically significant actions of leptin are also preserved. For example, in humans, the response of obese subjects to weight loss is fundamentally intact [62], suggesting that the ‘extra’ leptin in the context of obesity is able to exert relevant biological effects on other mechanisms besides those involved in the control of feeding.

To date, most data concerning the cellular and molecular mechanisms of obesity-associated leptin resistance have been obtained in experimental rodent models, including DIO, genetic models, obesity-prone models, early overfeeding and age-related obesity animals with hypothalamic lesions. In this context, we will focus briefly on the DIO model, the most frequent experimental model used to study leptin resistance, since it shares many characteristics with human obesity, including an attenuated response to the anorexigenic effect of leptin [59,63]. DIO models are obtained by feeding animals with hypercaloric diets, but dietary fats alone are insufficient to block the response to leptin, suggesting that hyperleptinaemia is required for the development of leptin resistance [64], which can be obtained within 8 days of high-dose leptin treatment [65]. Furthermore, rats chronically overexpressing central leptin initially responded to leptin gene delivery, then became leptin-resistant, and, on a high-fat diet, they consumed more energy, gained more weight and accumulated greater visceral fat mass than controls, suggesting that leptin resistance is both a consequence and a cause of obesity [66].

**Figure 2:**
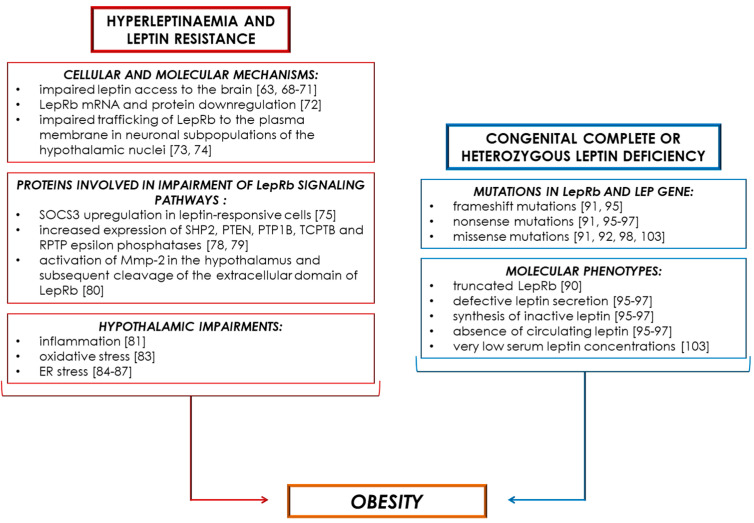
Schematic overview summarising obesity-associated hyperleptinaemia, leptin resistance and leptin deficiency.

Several cellular and molecular mechanisms of leptin resistance have been identified, and we will describe some of them henceforth (Figure 2). Leptin is transported intact from the blood to the brain through the blood–brain barrier (BBB) by a specific and saturable system [67]. Several studies have shown that the consumption of dietary fats induces the apoptosis of neurons and a reduction of synaptic inputs in the arcuate nucleus and lateral hypothalamus [68]. In addition, DIO models and New Zealand obese mice exhibit resistance to peripherally administered leptin, yet were responsive to chronic infusions of leptin intracerebroventricularly [63,69]. Meanwhile, leptin concentrations in cerebrospinal fluid were strongly correlated with plasma levels in a nonlinear manner and with BMI, suggesting that plasma leptin enters human cerebrospinal fluid in proportion to body adiposity [70]. However, the efficiency of this uptake (measured as the cerebrospinal fluid: plasma leptin ratio) in lean individuals was 4.3-fold greater than that in obese individuals [71]. Thus, all the data described above suggest that impaired leptin access to the brain is responsible for leptin resistance in obesity and further weight gain (Figure 1).

Other molecular mechanisms involved in the onset of leptin resistance involve hypothalamic LEPRs (LepRbs). Specifically, LepRb mRNA and protein downregulation [72], along with impaired trafficking of LepRb to the plasma membrane in neuronal subpopulations of the hypothalamic nuclei that control energy homeostasis [73,74], have emerged as novel mechanisms of leptin resistance.

Multiple molecules and proteins are involved in the impairment of LepR signalling pathways, thus contributing to obesity-associated leptin resistance and hyperleptinaemia. It is known that SOCS3 is able to block LepRb signalling and, since hyperleptinaemia is characterised by high hypothalamic SOCS3 levels, it was hypothesised that the upregulation of SOCS3 in leptin-responsive cells is, therefore, a potential mechanism for leptin resistance, a characteristic feature of human obesity [75]. Studies in mice lacking SOCS3 proteins, specifically in LEPR-expressing cells (*LepR SOCS3 knockout* (KO)) [76] and in transgenic mice overexpressing SOCS3 in either POMC or LEPR-expressing neurons at levels similar to what is observed in DIO models [77], confirmed the key role of SOCS3 in leptin sensitivity. Leptin action may also be regulated by protein phosphatases, and, to date, five main phosphatases involved in leptin signalling have been identified: SHP2, PTEN, PTP1B and the recently implicated TCPTP and RPTP epsilon [78]. With the exception of SHP2, which promotes leptin signalling by coupling to ERK kinase, all of the other four phosphatases work by inhibiting leptin signalling, leading to leptin resistance [78]. Furthermore, increased expression of these phosphatases has also been shown to promote leptin resistance [79]. Recently, another molecular mechanism was suggested for leptin resistance through the activation of matrix metalloproteinase-2 (Mmp-2) in the hypothalamus and subsequent cleavage of the extracellular domain of the LEPR [80]. The deletion of Mmp-2 allows for the restoration of LEPR expression and the reduction of circulating leptin concentrations in obese mice [80].

In addition, hypothalamic impairments in terms of inflammation, oxidative stress and endoplasmic reticulum (ER) stress might contribute to the development of leptin resistance. It has been demonstrated that the high consumption of sugar and saturated fat induces an inflammatory response in the hypothalamus, in turn promoting the development of central leptin resistance and obesity. Specifically, this inflammatory signalling involves changes in the expression and activity of several proteins, such as Toll-like receptor 4, IκB kinase-β/nuclear factor-κB, c-Jun N-terminal kinase and SOCS3 and proinflammatory cytokines, as well as the induction of ER stress and autophagy defects [81]. Furthermore, chronic low-grade inflammation within the hypothalamus might also represent a possible mechanism for central leptin resistance not only in obesity but also in polycystic ovarian syndrome, as seen in murine models [82]. Additionally, the hypothalamus of obese subjects was also found to be characterised by the presence of oxidative stress, which leads to the depletion of POMC neurons, and, consequently, to the induction of systemic leptin resistance and obesity [83]. In addition, ER stress, caused by an excessive accumulation of unfolded proteins, and able to activate the unfolded proteins response (UPR), may contribute to an impairment in leptin signalling [84]. Indeed, ER, stress-induced pharmacologically by using tunicamycin, thapsigargin or brefeldin A was able to block leptin-induced hypothalamic STAT3 phosphorylation and to augment appetite and body weight gain in mice, whereas chemical chaperones, 4-phenyl butyric acid and tauroursodeoxycholic acid, which have the ability to reduce ER stress, acted as leptin-sensitising agents, thus providing the basis for potential novel treatments of obesity [85,86,87].

## 5. Obesity-Associated Leptin Deficiency

Although most forms of human obesity are polygenic and multifactorial, there are also several rare cases of obesity caused by leptin deficiency [88]. These disturbances can be completely reversed by leptin administration [89], as described below. It is possible to distinguish both complete congenital leptin deficiency, defined as a recessive genetic disorder associated, from severe early-onset obesity heterozygous leptin deficiency, whose estimated prevalence is only up to 5–6% of total obese individuals. Moreover, mutations may be related to the *LEPR* gene or to the *LEP* gene [88], and the latter is more common than the former [18] (Figure 2).

In regard to the *LEPR* gene, Clément et al. described a homozygous mutation in the human *LEPR* gene that resulted in a truncated *LEPR* lacking both the transmembrane and intracellular domains and, in addition to their early-onset morbid obesity, patients homozygous for this mutation had no pubertal development, with reduced growth hormone and thyrotropin levels, suggesting that leptin is an important physiological regulator of several endocrine functions in humans [90]. Additional homozygous frameshift, nonsense and missense *LEPR* mutations have been identified in severely obese patients from consanguineous families [91,92]. Additionally, other novel *LEPR* mutations were detected both in two unrelated girls with severe obesity [93] and in obese children from inbred Pakistani families, which constitute 3% of the whole cohort of severely obese children [94].

Mutations in the *LEP* gene might foster nonsense-mediated mRNA decay, defective leptin secretion, synthesis of inactive leptin or absence of circulating leptin [95,96,97]. The first evidence of congenital leptin deficiency in humans was provided by two severely obese children who are cousins within a highly consanguineous family of Pakistani origin and whose serum leptin levels were very low despite their markedly elevated fat mass [95]. In addition, both subjects showed a homozygous frameshift mutation involving the deletion of a single guanine nucleotide in codon 133 of the gene for leptin, which resulted in a truncated protein that was not secreted [95]. Strobel et al. identified a homozygous missense mutation in the *LEP* gene in three adults belonging to a family of Turkish origin [98]. To date, many other mutations have been reported in the *LEP* gene in consanguineous families [99,100,101,102]. For example, Yupanqui-Lozno et al. recently reported a novel homozygous missense mutation in *LEP* associated with very low serum leptin concentrations, hyperphagia and early-onset obesity in two severely obese sisters from Colombia born from consanguineous parents [103]. The analysis of serum leptin level is a useful test in patients with severe early-onset obesity, but it is also plausible that mutations in the *LEP* gene could result in a bioinactive form of the hormone in the presence of apparently appropriate leptin levels. Further investigation is therefore needed for this type of analysis.

As described in detail above, leptin may act to control both energy homeostasis and thermoregulation and could be one of the mediators that can activate BAT and thermogenesis. Therefore, an important question concerns the role of BAT metabolism under conditions of leptin deficiency. Martins et al. focused on BAT from *ob/ob* mice demonstrating impaired thermogenic signalling (β3-AR, PGC-1α and UCP-1) in association with reduced expression of fatty acid synthesis–related genes (*SREBP1c* and *FAS*), reduced fatty acid mobilisation–related genes (*CD36*, *FABP4* and *perilipin*) and reduced fatty acid oxidation genes (*CPT1*) [104,105]. In addition, BAT in *ob/ob* mice was also characterised by altered insulin signalling (pAKT, TC10 and GLUT4) and gene markers of local inflammation (*IL-1β*, *IL-6*, *TNF-α* and *MCP1*) [104,105], suggesting that the lack of substrate for thermogenesis and local inflammation both negatively regulated thermogenic signalling in *ob/ob* mice. In addition, *ob/ob* mice regained the lost weight, by transient caloric restriction, to the level of ad libitum controls [106]. They did so by reducing EE, but, unlike wild-type mice, there was no compensatory relative hyperphagia, suggesting that non leptin-dependent mechanisms were involved in regulating the body weight of leptin-deficient mice [106].

The clinical phenotypes associated with leptin and LEPR deficiencies are very similar [91,95,107]. Patients show normal birth weight, then exhibit rapid weight gain in the first few months of life, up to severe obesity. In addition, body composition measurements highlighted that these disorders are characterised by the preferential deposition of fat mass, with excessive amounts of subcutaneous fat present over the trunk and limbs [108]. Children with leptin deficiency also show high rates of infection due to abnormalities in the number and function of T lymphocytes [101]. In those who survive, obesity continues into adult life, with hepatic steatosis, hyperinsulinaemia and the development of type 2 diabetes occurring in the third or fourth decade [98,109].

LepRb: leptin receptor; Mmp-2: matrix metalloproteinase-2; PTEN: Phosphatase and tensin homolog; PTP1B: protein–tyrosine phosphatase 1B; RPTP epsilon: receptor-type form of protein tyrosine phosphatase epsilon; SHP2: Src homology region 2 domain-containing phosphatase-2; SOCS3: suppressor of cytokine signalling 3.

## 6. Pharmacotherapy of Leptin Signalling

As previously discussed, leptin has been recently come to be considered a ‘thermolipokine’ because it may support both thermogenic and browning responses. In addition, activation of BAT in humans is associated with marked improvements in metabolic parameters since it is an important organ for thermogenesis with the capacity to induce energy-consuming futile cycles [110]. For these reasons, BAT is emerging as an interesting and promising target for therapeutic intervention in obesity and metabolic disease, and leptin-induced thermogenesis could be designed as a new promising strategy to counteract obesity and related metabolic derangements.

Leptin is known to exert control of body weight and energy homeostasis via central mechanisms. However, leptin signalling undergoes an impairment under chronic leptin overproduction, as previously described [64]. This condition induces body weight gain [111] and impaired thermogenesis [112]. Similarly, metabolic failures and obesity features have also been observed when leptin deficiency develops in adulthood, highlighting that the lack of leptin can promote obesity even though its deficiency is not congenital [111]. Therefore, both higher and lower leptin levels represent a metabolically unfavourable condition, and this adipose-derived hormone could thus exert beneficial effects only when it acts in a tight physiological range of concentrations.

As previously mentioned, the first consequence of hyperleptinaemia is leptin resistance. In this regard, the prevention and reversion of leptin resistance may represent an important challenge in the field of obesity treatment; yet, the main results to date are derived from animal models. Focusing on improvements in metabolism and body weight and the recovery of leptin sensitivity obtained from in vivo blunted deletion of leptin [111], Zhao et al. investigated a novel anti-obesity strategy consisting of treatment with human anti-leptin antibodies (hLep2, hLep3, hLep5). Among these, hLep3 displayed a powerful weight-lowering efficacy, enhanced glucose tolerance and improved insulin sensitivity in obese mice (Table 1) [111]. Moreover, following the infusion of hLep3, leptin-resistant obese mice showed an increase in EE, a reactivation of thermogenic programming in brown fat (i.e., ↑UCP-1 and PGC-1α) and a reduction in hypothalamic expression of key markers of inflammation and central leptin resistance (i.e., SOCS3, TNF-α, IL-1β) [111]. Hence, these results indicate that the re-sensitisation of leptin signalling mediated by neutralising antibodies has a promising weight-lowering and thermogenic efficacy, thus making it a potential therapy against leptin-resistant obesity.

Some evidence supports the idea that central leptin resistance develops when leptin transport efficiency across the BBB is compromised rather than when hypothalamic leptin insensitivity begins (Figure 1) [58,113]. One of the therapeutic approaches favouring the recovery of BBB functions, whose permeability is compromised under obesity conditions [113], is represented by angiotensin II receptor blockers, whose administration ameliorates body weight and the metabolic profile [114,115,116]. Among these, telmisartan (TEL) was shown to exert favourable metabolic benefits by preventing hypothalamic inflammation [117] and by enhancing leptin transport through the BBB [118], thus restoring leptin sensitivity [115] and counteracting the development of obesity under excess nutrient intake [118]. Additionally, TEL is known to evoke several metabolic responses in obese mice with the improvement of insulin resistance and increased insulin-induced glucose uptake by adipose tissue (WAT and BAT), as it retains PPAR-δ agonism effects [119] and sustained nonshivering thermogenesis via sympathetic control (Table 1) [120]. Furthermore, chronic in vivo exposure to TEL decreased adipogenesis and upregulated the expression of a key thermogenic mediator (UCP-1) in adipose tissue [119]. The mechanisms by which TEL induces a thermogenic response are still unclear. We can hypothesise, however, that the restoration of BBB permeability and leptin uptake from the brain after TEL administration might favour the recovery of central leptin signalling, resulting in downstream adaptive thermogenesis. Whether this drug may exert similar metabolic effects in humans has yet to be demonstrated. Recent clinical research has proved the well-known cardiometabolic amelioration of TEL-based therapy in obese patients, even though no changes in body weight occurred [121]. However, after TEL treatment, the adipose tissue responded by increasing the adiponectin secretion and by reducing leptin release [122]. Therefore, the use of angiotensin receptor blockers on obesity in a clinical setting could restore central leptin sensitivity, whose downstream results could enhance BAT activity.

Another hallmark of leptin resistance is low-grade inflammation within the hypothalamus [81]; hence, pharmacological mitigation of these processes could be a new way to restore leptin sensitiveness. Corroborating results showed that hypothalamus inflammation is associated with impaired leptin signalling and plasminogen activator inhibitor-1 (PAI-1) expression [123,124], whose activity was recently extended in metabolism regulation [125,126]. Elevated circulating levels of PAI-1 were found under obesity conditions [127] and also appeared to have a predictive role for metabolic syndrome [127]. Particularly, this factor exacerbates adipose tissue dysfunction, worsening both inflammatory and metabolic dysregulation [128]. Hence, in vivo disruption of PAI-1 protects against obesity in part via enhanced EE [129]. Indeed, increased body weight due to energy overload was suppressed following the inhibition of PAI-1 by M5441 in association with an upregulation of thermogenic genes (i.e., *UCP-1*, *DIO-2*, *CIDEA*, *PRDM-16*) in brown depots [126] and amelioration of lipid turnover [130]. In this scenario, decreased plasma leptin levels were observed, suggesting that the metabolic benefits associated with PAI-1 inhibition could be due to the recovery of leptin sensitivity [126]. Obese mice pre-treated with M5441 responded to leptin infusion with sustained suppression of body weight gain as compared with untreated mice, which showed a weak leptin sensitiveness (Table 1) [126]. Nowadays, the paucity of human studies considering the anti-obesity effects of PAI-1 antagonism makes clinical application still a distant expectation. Data from in vivo research, however, allows us to highlight that PAI-1 antagonists might be an effective intervention to prevent the development of obesity and its sequelae by restoring leptin responsiveness with improved energy dissipation and thermogenic control.

Notably, the sensitiveness of leptin may also be regulated by indirect mechanisms. Compelling efforts reported that modifications of the gut microbiota could affect leptin sensitivity [131]. It is well-known that the contributions of gut microbiota in preserving healthy metabolism, as well as its alterations, can lead to the development of metabolic illness [132]. Several studies have discovered that feeding habits and certain natural components modify the intestinal microbial profile [133]. Particularly, in a recent study, it was shown that *Panax notoginseng saponins* (PNS) modulates the gut microbiota via an increased abundance of healthy bacterial species (i.e., *Akkermansia muciniphila*, *Parabacteroides distasonis*) mostly implicated in the regulation of energy homeostasis [7]. Moreover, PNS-based therapy-induced weight loss, increased EE and enhanced the expression of BAT thermogenic (i.e., *UCP-1*, *PGC-1α*, *DIO-2*) and browning genes (i.e., *UCP-1*, *PRDM-16*, *PGC-1α*) in DIO mice [7]. Nevertheless, this compound failed to promote these favourable metabolic effects in mice with leptin signalling abrogation, revealing that the presence of leptin resistance might interfere with PNS-based therapy [7]. Accordingly, this finding suggests that leptin signalling participates in the regulation of thermogenesis induced by modifications to the gut microbiota [7]. Indeed, WAT from mice treated with PNS showed increased phosphorylation of key mediators of a leptin-mediated thermogenic response (i.e., AMPK-α, STAT3), whose activity is essential for the leptin pathway function (Table 1) [7,134].

Therefore, lifestyle and dietary modifications could be a promising anti-obesity therapeutic to restore leptin signalling, similarly to what has been observed after PNS treatment. Whether PNS modifies the human gut microbiota and promotes thermogenesis through the leptin/AMPK-α/STAT3 axis is still unclear. Further investigations should elucidate the involvement of gut microbiota in leptin signalling control.

## 7. Pharmacological Treatment of Leptin Deficiency

Some genetic forms of obesity result from complete congenital leptin deficiency or heterozygous leptin deficiency [104]. Several data have demonstrated that leptin deficiency is entirely treatable with daily subcutaneous injections of recombinant human leptin [135,136]. The form of leptin that is available for human therapy is the recombinant methionyl human leptin (metreleptin), which is composed of 146 amino acids, as in the mature human leptin, with an additional methionyl residue at the N-terminal end of the recombinant protein [137]. It has been demonstrated that patients with congenital leptin deficiency treated with metreleptin showed profound weight loss, increased physical activity, less hunger and desire to eat, less food intake, greater fullness both before and after meals, and changes in endocrine function and metabolism, including the resolution of type 2 diabetes mellitus and hypogonadism. These results highlight the role of the leptin pathway in adults with key effects on the regulation of body weight, gonadal function behaviour [135]. In regard to EE, Galgani et al. demonstrated that, before weight loss, subjects with congenital leptin deficiency and control subjects had similar EE profiles, while, after weight loss (approximately 15 kg), control subjects had EE levels lower than expected for their new weight and body composition, whereas leptin-treated subjects presented EE values that were not different from the reference population. In addition, before weight loss, fat oxidation was similar between groups, and, after weight loss, leptin-treated subjects had higher fat oxidation than both controls and the reference population [136]. Collectively, these data highlighted that, in congenital leptin-deficient subjects, leptin replacement enhanced energy metabolism by partially preventing the reductions in metabolic rate and fat oxidation so often observed during energy restriction. Similar results on leptin replacement-induced weight loss were obtained in other studies where leptin therapy did not increase EE [101,137]. Of note, metreleptin also showed metabolic effects (e.g., decrease in triglycerides and increase in high-density lipoprotein cholesterol [101,109,135]), endocrine effects (i.e., increased white blood cell count and T-cell responsiveness [101,138]) and neuroimaging changes (i.e., decreased activation of regions linked to hunger and increased activation of regions linked to inhibition and satiety [139]) in humans with congenital leptin deficiency due to mutations in the *LEP* gene. In the future, clinical trials will be needed to evaluate whether leptin therapy may also be effective in individuals with a partial genetic deficiency in leptin [139].

Since leptin shares its signalling pathway with other hormones involved in energy metabolism regulation, combined therapies might provide significant metabolic improvements. The pancreatic hormone amylin (a short-term satiety signal) is one hormone of interest [140,141,142], and, in a rodent model of obesity, the combined administration of amylin with leptin elicited synergistic effects on body weight and markedly reduced adiposity [143]. Clinical evidence to support integrated neurohormonal therapy for obesity was also obtained in overweight/obese humans where combination treatment with pramlintide, an amylin analogue, and metreleptin induced greater weight loss than either agent alone [144,145].

Recent studies have demonstrated that melatonin receptor type 1 signalling played an important role in maintaining metabolic homeostasis and was an important modulator of leptin signalling, while its removal led to leptin and insulin resistance [146]. Specifically, its anti-obesity properties were evaluated in *ob/ob* mice, where the administration of melatonin induced significant weight loss and reduced adipose tissue inflammation by restoring the physiological adipokine patterns (i.e., reduced TNF-α, resistin, visfatin) [147]. In addition, chronic melatonin treatment in rats behaved as an inducer of white fat browning with thermogenic properties (i.e., increased UCP-1 and PGC-1α), underling the anti-obesity effect of melatonin as well as its antidiabetic and lipid-lowering properties (Table 1) [148]. Given these promising results, it may be useful to consider the possibility of also using melatonin in pathological conditions of leptin deficiency.

In addition, it has been demonstrated that treatment with a melanocortin receptor agonist is effective to counteract obesity and related metabolic defects both in leptin-resistant (e.g., DIO models) and leptin-sensitive (*ob/ob*) mouse models of obesity, and that its effects on food intake and body weight are more pronounced in DIO mice than in lean mice [149]. Recently, results from phase III trials support setmelanotide, a specific melanocortin receptor agonist, for the treatment of severe obesity and hyperphagia caused by POMC or LepRb deficiency [150]. In this multicentre study, the treatment of setmelanotide was associated with significant weight-loss reductions in hunger scores and a good safety profile, supporting its potential long-term use as a treatment for early-onset severe obesity and hyperphagia caused by POMC or LEPR deficiency.

**Table 1:** In vivo and in vitro effects of potential therapeutic strategies of obesity-related hyperleptinaemia and leptin deficiency.

Drugs	Species	In Vitro	In Vivo/Ex Vivo	Effects
***For Hyperleptinaemia***				
*hLep3 antibodies*	Mouse	-	↑ *UCP-1* and *PGC-1α* mRNA in WAT and BAT [111] ↓ *SOCS3*, *TNF-α* and *IL-1β* mRNA in the hypothalamus [111]	↑ Glucose tolerance [111] ↑ EE [111] ↓ Body weight [111] ↓ Hypothalamus inflammation [111] ↑ Leptin sensitivity [111]
*Telmisartan*	Mouse	↑ PPAR-δ signalling [119] ↓ Adipogenesis [119]	↑ UCP-1 protein in BAT [119]	↑ Leptin sensitivity [115] ↓ Hypothalamus inflammation [117] ↑ Leptin transport through the BBB [118] ↓ Body weight [118] ↓ Insulin resistance [120] ↑ Sympathetic nervous system [120]
	Human	-	-	↑ Cardiac function [121] ↓ Plasma leptin levels [122]
*PAI-1 inhibitor M5441*	Mouse	-	↑ *UCP-1*, *DIO-2*, *CIDEA* and *PRDM-16* mRNA in BAT [126]	↑ Leptin sensitivity [126] ↓ Plasma leptin levels [126] ↓ Body weight [126] ↑ Lipolysis [130]
*Panax notoginseng saponins*	Mouse	-	↑ UCP-1, PGC-1α and DIO-2 mRNA/protein in BAT [7] ↑ UCP-1, PRDM-16 and PGC-1α mRNA/protein in WAT [7] ↑ AMPK-α/STAT3 signalling in WAT [7]	↑ EE [7] ↓ Body weight [7]
***For Leptin Deficiency***				
*Metreleptin*	Human	-	-	↓ Triglycerides [101] ↑ HDL [101] ↑ T-cell responsiveness [101] ↓ Body weight [135] ↑ Physical activity [135] ↓ Hunger [135] ↑ Fat oxidation [136] ↓ Fall in EE during caloric restriction [136]
*Metreleptin/pramlintide*	Mouse	-	-	↓ Body weight [143]
	Human	-	-	↓ Body weight [144]
*Melatonin*	Mouse	-	↓ TNF-α, resistin and visfatin proteins in adipose tissue [147] ↑ UCP-1 and PGC-1α proteins in WAT [148]	↓ Body weight [147] ↓ Adipose tissue inflammation [147]
*Melanocortin receptor agonists*	Mouse	-	-	↓ Body weight [149] ↓ Food intake [149]
	Human	-	-	↓ Body weight [150]

↑, increase; ↓, decrease; -, not available. Uncoupling protein 1 (UCP-1), peroxisome proliferator-activated receptor gamma coactivator 1-alpha (PGC-1α), white adipose tissue (WAT), brown adipose tissue (BAT), suppressor of cytokine signalling 3 (SOCS3), tumour necrosis factor alpha (TNF-α), interleukin-1beta (IL-1β), type II iodothyronine deiodinase (DIO-2), cell death activator (CIDEA), PR domain–containing 16 (PRDM16), AMP-activated protein kinase alpha (AMPK-α), signal transducer and activator of transcription 3 (STAT3).

## 8. Conclusions

Leptin is a pleiotropic peptide hormone produced predominantly by adipocytes, released into the bloodstream and crucial for the neuroendocrine control of energy homeostasis. Obesity might also be associated with hyperleptinaemia, which reflects a state of leptin resistance involving leptin and molecular pathways downstream of LEPR, or with leptin deficiency, which might be either complete or heterozygous (Figure 1 and Figure 2). Impairment of leptin signalling in neuronal populations of hypothalamic nuclei that control energy balance leads to increased food intake, reduced EE, insulin resistance and adipose tissue expansion. Indeed, recent studies showed that re-sensitisation of leptin signalling mediated by neutralising antibodies restores both leptin and insulin sensitivity, promoting weight lowering and thermogenic activation and thus constituting a promising therapy against leptin resistance in obesity.

In addition, treatment of perturbations of leptin signalling with LEPR agonists observed in obese patients with leptin deficiency was associated with significant weight loss and reduction in hunger scores after 1 year of treatment. Strategies aimed at restoring leptin sensitivity in hypothalamic neurons might represent a hopeful approach for the treatment of obesity and associated comorbidities.

Lastly, in view of the critical role of leptin in regulating thermogenesis via central control and the presence of BAT and beige adipocytes also in adult humans, a potential role of leptin as a possible new thermolipokine could be envisioned. However, the direct effects of leptin on BAT activity are still poorly explored, even though some reports observed the capability to induce browning. Nevertheless, based on current evidence, the action of leptin on thermogenesis appear to occur largely via an indirect central response.

Therefore, in the context of obesity and hyperleptinaemia, a better understanding of the mechanisms that impair leptin-mediated adaptive thermogenesis may facilitate the development of drugs able to promote a re-sensitisation of leptin signalling, with the ultimate aim of enhancing thermogenesis, favouring body weight reduction resolving obesity-associated metabolic disorders (Table 1).
